# Actor Placement Optimization in WSANs by the PSO-HC-DGA Hybrid System for Two-Zone Industrial Environments

**DOI:** 10.3390/s26051471

**Published:** 2026-02-26

**Authors:** Paboth Kraikritayakul, Admir Barolli, Shinji Sakamoto, Shunya Higashi, Phudit Ampririt, Leonard Barolli

**Affiliations:** 1 Department of Information and Communication Engineering, Fukuoka Institute of Technology, Fukuoka 811-0295, Japan; bd25103@bene.fit.ac.jp; 2 Department of Information Technology, Faculty of Information Technology, Aleksander Moisiu University of Durres, 2009 Durrës, Albania; admirbarolli@uamd.edu.al; 3 Department of Information and Computer Science, College of Engineering, Kanazawa Institute of Technology, Nonoichi 921-8501, Japan; shinji.sakamoto@ieee.org; 4 Department of Information Engineering, Oita National Colleges of Technology, Oita 870-0152, Japan; p-ampririt@oita.kosen-ac.jp

**Keywords:** Wireless Sensor and Actor Networks, actor placement optimization, hybrid metaheuristics, real-coded crossovers, industrial two-zone topology

## Abstract

Wireless Sensor and Actor Networks (WSANs) are critical for industrial automation in the context of Industry 4.0, yet the optimal placement of actors to ensure connectivity and coverage remains an NP-hard problem. This study addresses the Actor Placement Problem (APP) in constrained, two-zone industrial environments. We propose a hybrid system, the PSO-HC-DGA hybrid system, which integrates Particle Swarm Optimization (PSO), Hill Climbing (HC), and the Distributed Genetic Algorithm (DGA). We evaluate four crossover methods (UNDX, SPX, BLX-α, and psBLX) combined with two actor replacement methods (RIWM and FC-RDVM) for small-, medium-, and large-scale scenarios. The simulation results demonstrate that psBLX is the most effective of the four crossover methods. In the small-scale scenario, it achieved better load balancing combined with RIWM, while in the medium-scale scenario, psBLX achieved full sensor coverage with RIWM and good load balancing with FC-RDVM. For the large-scale scenario, we compared the performance of the implemented hybrid system with that of a PSO system. The hybrid system showed 100% connectivity and achieved better sensor coverage than the PSO system. The Kruskal–Wallis test confirmed that the performance differences in load balancing were statistically significant. We conclude that the proposed hybrid system using psBLX enables robust and high-performance deployment in two-zone industrial WSANs.

## 1. Introduction

The advent of the Fourth Industrial Revolution (Industry 4.0) has led to a paradigm shift in manufacturing and automation [1,2], driven largely by the proliferation of the Industrial Internet of Things (IIoT). Within this ecosystem, Wireless Sensor and Actor Networks (WSANs) have emerged as a pivotal technology, extending the passive monitoring capabilities of traditional Wireless Sensor Networks (WSNs) by incorporating actor nodes capable of performing physical actions and thus interacting with the environment [3]. Actors possess richer power resources and greater processing capabilities than sensors, allowing them to execute complex tasks such as valve regulation, robotic actuation, and disaster mitigation [4]. However, the efficacy of a WSAN is determined by the topological arrangement of nodes. The Actor Placement Problem (APP) is a critical design challenge. In complex industrial environments, particularly those characterized by distinct operational zones (e.g., separating hazardous processing areas from safe control centers), random or uniform deployment strategies often fail. They result in coverage holes, disconnected subnetworks, and uneven load balancing, which can lead to premature network failure [5].

Current research classifies the APP as an NP-hard problem [6]. Many research efforts have been dedicated for solving node placement problems in WSANs [7,8,9], and the research field is moving toward metaheuristic algorithms. Evolutionary algorithms, such as Genetic Algorithms (GAs) and Particle Swarm Optimization (PSO), have shown promising results in navigating the non-linear search spaces of network deployment [10]. Despite these advancements, standard single-population metaheuristics often struggle to balance the trade-off between exploration (searching new areas) and exploitation (refining existing solutions). PSO puts the network at risk of premature convergence to local optima [11], while GAs may suffer from slow convergence [12]. Furthermore, diverging hypotheses exist regarding the most effective mechanisms for solution recombination: while some studies shows that operators such as Blend Crossover (BLX-α) have good performance [13], others suggest that geometric, multi-parent operators such as Unimodal Normal Distribution Crossover (UNDX) or Simplex Crossover (SPX) are superior for high-dimensional dependencies [14,15].

To address these challenges, this research proposes a hybrid system called PSO-HC-DGA, which combines PSO, HC, and DGA, for robust deployment of actors in constrained, two-zone industrial topologies by integrating the global search parallelization of the Distributed Genetic Algorithm (Island Model) [16] with the rapid convergence of PSO and the local refinement capabilities of Hill Climbing (HC). By performing a comparative analysis of four crossover methods (UNDX, SPX, BLX-α and Parallelotope-Shaped Blend Crossover (psBLX)) and two actor replacement methods (the Random Inertia Weight Method (RIWM) and the Fast Convergence Rational Decrement of $V_{max}$ Method (FC-RDVM)), we elucidate the impact of operator selection on network performance.

The simulation results demonstrated that in a small-scale scenario, all methods achieved full actor connectivity and complete sensor coverage. Considering load balancing, the evaluation results showed that **RIWM** combined with **psBLX** [17] achieved superior load balancing. For a medium-scale scenario, all combinations maintained full actor connectivity but resulted in different sensor coverage. **RIWM** combined with **psBLX** covered all sensors, while **FC-RDVM** combined with **psBLX** had better load balancing. We performed the Kruskal–Wallis H-test, which confirmed that the observed differences in load balancing were statistically significant. The H-statistics increased with problem size, indicating that the choice of crossover method becomes increasingly critical as network complexity grows. For a large-scale scenario, we compared the performance of the hybrid system with that of a PSO system. The hybrid system showed robust scalability, maintaining 100% actor connectivity and achieving high sensor coverage, while the PSO system failed to establish a WSAN.

The remainder of this paper is organized as follows. Section 2 introduces the fundamental concepts of WSANs and the challenges of actor placement. Section 3 describes the intelligent algorithms utilized in this study, including PSO, HC and DGA. Section 4 details the proposed PSO-HC-DGA hybrid system, outlining its architecture and the multi-objective fitness function. Section 5 presents the simulation results for small-, medium-, and large-scale scenarios. Finally, Section 6 offers the conclusion and suggests directions for future research.

## 2. Wireless Sensor and Actor Networks (WSANs)

**Figure 1:**
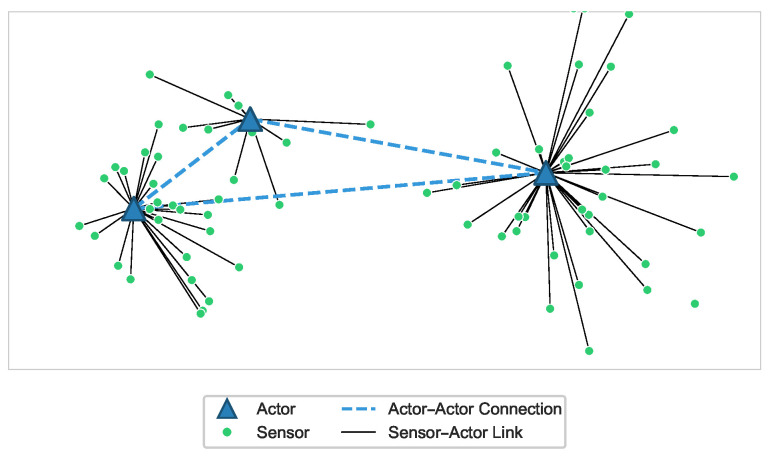
An Example of Wireless Sensor and Actor Networks (WSANs).

WSANs integrate distributed sensing with networked actuation, enabling the detection of events and the timely execution of actions within the physical environment. In typical deployments, sensors collect measurements such as temperature, vibration or position, while actors perform tasks such as opening industrial valves, moving mobile platforms or activating safety mechanisms. Actors possess superior computational and communication capabilities compared to sensors and collaborate to achieve application objectives in domains such as industrial automation and cyber-physical systems [3,18]. Unlike traditional WSNs, WSANs introduce additional challenges stemming from the coexistence of sensing and actuation, including timeliness, reliability and coordination among multiple actors as well as between sensors and actors [3,18]. Figure 1 provides an illustrative example, where blue triangles represent actors and green points represent sensors. Solid black lines signify wireless connectivity between sensors and actors, indicating successful sensor–actor connections. The dashed blue lines represent actor–actor connectivity, forming the backbone of the WSAN and ensuring reachability between zones.

In this study, we consider industrial facilities organized into two operational zones (e.g., production and storage areas). Such sites necessitate reliable data transmission from sensors to decision points and from decision points to actors, subject to practical constraints on radio range, interference and energy consumption on the sensor side. Technology choices for IIoT further influence design trade-offs [19], which ultimately depend on the physical layout and interference conditions [20]. Within this context, WSANs present an attractive solution because of their incremental deployment capabilities, adaptability to evolving workflows and support for closed-loop control.

### 2.1. Actor Placement Problem

The placement of actors is a crucial design decision for WSANs. Given a known or sampled sensor distribution across two industrial zones, wireless constraints, and a budget on the number of actors, the objective is to determine actor locations that ensure reliable operation. Inadequate placement can fragment actor interconnectivity, expose sensors and concentrate workload on a limited number of actors, thereby compromising reliability and increasing delay. Owing to the continuous and highly combinatorial nature of the placement search space, characterized by numerous local optima, the problem is regarded as NP-hard [21,22]. Consequently, prior research on actor selection and connectivity restoration has motivated the adoption of heuristic and metaheuristic optimization in realistic deployments.

**Figure 2:**
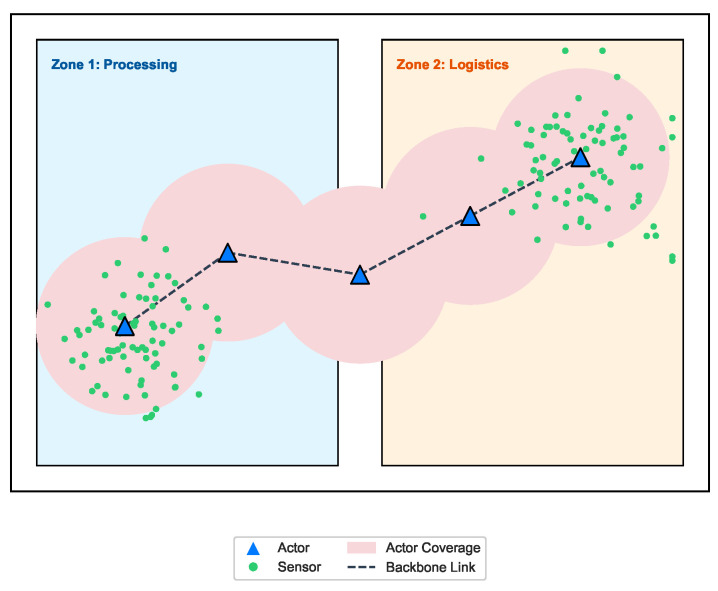
Two-zone industrial topology.

In our work, we investigate two-zone industrial environment for small and medium scales, as depicted in Figure 2, where zone 1 can be used for processing and zone 2 for logistics. We compare crossover and actor replacement methods within our hybrid system to understand how different methodological choices affect connectivity, coverage and load balancing across various scales and scenarios.

### 2.2. Challenges in Two-Zone Topologies

Dividing an industrial environment into distinct functional zones, such as Zone 1 for processing and Zone 2 for logistics, aligns with realistic operational requirements and introduces significant heterogeneity that complicates standard actor placement strategies. We identify three challenges in two-zone environments.

- Inter-Zone Connectivity Bottleneck: The boundary between zones often serves as a logical or physical constraint point, creating a “weak link” in the network backbone. Standard placement algorithms that assume a homogeneous field tend to distribute actors uniformly, leading to a sparse distribution at the boundary interface. This vulnerability arises when the few actors bridging two zones fail or move to service a local event, causing network partition and isolating Zone 1 from Zone 2. Robust connectivity restoration mechanisms that can operate across partitioned boundaries are essential to address this issue [5].
- Funneling Effect and Energy Holes: In a two-zone environment, data traffic flows in a specific direction from the sensor-dense and high-activity Zone 1 to the aggregation points in the safer and accessible Zone 2. Actors near the inter-zone boundary become dominant relays, forwarding aggregated traffic between the zones. This heavy relay burden causes rapid energy depletion for boundary actors, resulting in the “energy hole” problem. The premature death of these critical nodes disrupts network connectivity [23].
- Heterogeneous Density Requirements: Zones containing hazardous processes, such as Zone 1, demand redundant coverage (*k*-coverage) and low-latency response, necessitating a high density of actors. In contrast, storage zones, such as Zone 2, require only sparse monitoring. Single-objective optimization algorithms often struggle to balance these conflicting density requirements, either over-provisioning Zone 2 or under-provisioning Zone 1. This industrial application demands design principles that transcend simple uniform random deployment [24].

## 3. Intelligent Algorithms

### 3.1. Particle Swarm Optimization (PSO)

PSO is a population-based metaheuristic algorithm inspired by the social behavior of bird flocks and schooling fish [10]. PSO optimizes a problem by iteratively refining a candidate solution on the basis of a predefined fitness function. In the context of WSAN actor placement, each “particle” represents a potential solution, comprising a set of coordinates for all actor nodes within the network. The swarm navigates the search space, guided by both its own local best known position and the best known position discovered by the entire swarm.

Let $N_{p}$ represent the number of particles in the swarm. Each particle *i* (where $i\;=\;1,\ldots ,N_{p}$) is characterized by the position vector $x_{i}$ and the velocity vector $v_{i}$ within the *D*-dimensional search space. The movement of these particles is governed by two primary equations: the velocity update and the position update.

**Figure 3:**
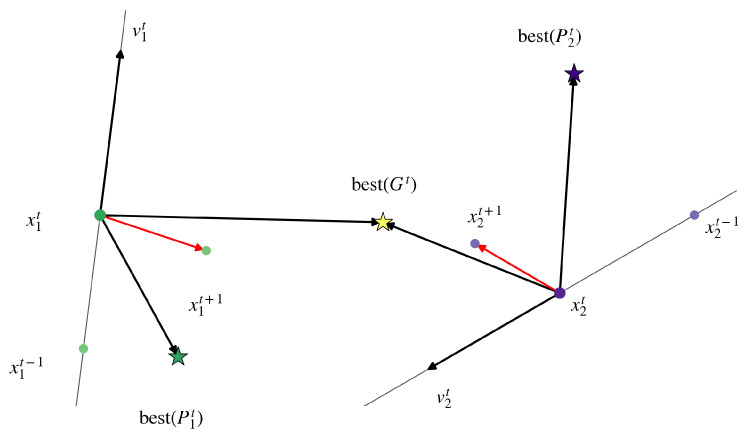
Vector diagram illustrating the velocity and position update mechanisms in PSO.

Figure 3 illustrates the movement process for two particles, $x_{1}^{t}$ and $x_{2}^{t}$, at iteration *t*. The new velocity vector for each particle, represented by the red arrow ($v^{t+1}$), is the sum of three component vectors corresponding to the terms in the velocity equation. For particle $x_{1}^{t}$, these components include the inertia component, which preserves the particle’s previous motion direction ($v_{1}^{t}$); the cognitive component, which guides the particle towards its personal best position ($\mathrm{best}(P_{1}^{t})$); and the social component, which attracts the particle towards the best position discovered by the swarm ($\mathrm{best}(G^{t})$). The resultant velocity $v_{1}^{t+1}$ then determines the particle’s new position $x_{1}^{t+1}$. A similar process is depicted for particle $x_{2}^{t}$, demonstrating how individual experiences, combined with a shared global objective, shape their respective trajectories.

At each iteration *t*, the velocity of particle *i* undergoes an update considering its inertia, its personal experience and the collective experience of the swarm. The velocity update equation is expressed as follows:

(1) $$v_{i}^{t+1}=\omega v_{i}^{t}+c_{1}r_{1}\left(\mathrm{best}\left(P_{i}^{t}\right)-x_{i}^{t}\right)+c_{2}r_{2}\left(\mathrm{best}\left(G^{t}\right)-x_{i}^{t}\right).$$

Once the velocity is determined, the position of the particle is updated for the next iteration:

(2) $$x_{i}^{t+1}=x_{i}^{t}+v_{i}^{t+1}.$$

The parameters of the two equations above are defined as follows:

- $v_{i}^{t}$ is the velocity of particle *i* at iteration *t*.
- $x_{i}^{t}$ is the current position of particle *i* at iteration *t*.
- ω is the inertia weight, which controls the impact of the previous velocity on the current one. It balances the trade-off between global exploration and local exploitation.
- $\mathrm{best}(P_{i}^{t})$ is the personal best position (pbest) reached by particle *i* up to iteration *t*.
- $\mathrm{best}(G^{t})$ is the global best position (gbest) found by any particle in the entire swarm up to iteration *t*.
- $c_{1}$ is the cognitive coefficient, which quantifies the particle’s tendency to return to its own best position.
- $c_{2}$ is the social coefficient, which quantifies the particle’s tendency to move toward the swarm’s best position.
- $r_{1},r_{2}$ are random values uniformly distributed in the range [0,1], introducing stochastic diversity to the search process.

By iteratively applying Equations (1) and (2), the swarm converges toward optimal actor placements that maximize the fitness function.

To further enhance the search behavior and adaptability of PSO in WSANs, we integrate specific actor replacement methods into the standard framework. These methods modify the fundamental way actors update their positions within the solution space [25]. In this study, we implement two actor replacement methods: RIWM and FC-RDVM.

#### 3.1.1. Random Inertia Weight Method (RIWM)

In RIWM, the inertia weight ω is randomly varied within a predefined range of 0.5≤ω≤1.0 at each iteration [26,27]. This randomization introduces stochasticity into the velocity update process. By dynamically adjusting the momentum, RIWM promotes both exploration and exploitation, thereby preventing the swarm from prematurely converging.

#### 3.1.2. Fast Convergence Rational Decrement of $V_{max}$ Method (FC-RDVM)

FC-RDVM aggressively reduces the maximum velocity ($V_{max}$) permitted for particles to promote faster convergence. This strategy facilitates the rapid settling of particles into promising regions of the solution space, making it effective in problems where fine-grained tuning is prioritized over broad exploration in subsequent iterations.

The value of $V_{max}$ at iteration *n* is calculated using Equation (3):

(3) $$V_{max}\left(n\right)=\sqrt{W^{2}+H^{2}}\times \frac{N-n}{N+\delta n},$$

where the variables are defined as follows:

- *W* and *H* represent the width and height of the search space.
- *N* is the maximum number of iterations.
- *n* is the current iteration.
- δ is a constant factor that controls the rate of velocity decrease.

### 3.2. Hill Climbing (HC)

Hill Climbing (HC) is a local search algorithm employed in optimization problems to enhance candidate solutions. It begins with an initial solution and iteratively explores neighboring configurations by making localized adjustments. If a neighboring solution offers an improvement in the objective function, it replaces the current solution. Otherwise, the search concludes in the current state. HC’s greedy nature enables rapid convergence with low computational cost.

Let $x_{t}$ represents the current solution at iteration *t*, and let f(x) denotes the objective function. A neighboring solution x’ is generated from the neighborhood of $x_{t}$ (Equation (4)). The update rule is governed by the following greedy criterion:

(4) $$x_{t+1}=\left\{\begin{array}{cc} x^{\prime } & \mathrm{if}\;f\left(x^{\prime }\right)>f\left(x_{t}\right)\\ x_{t} & \mathrm{otherwise} \end{array}\right..$$

In the context of WSAN optimization, HC is often employed to refine node positions by displacing actors within a small fixed radius and evaluating improvements in connectivity, coverage, and load balancing. For instance, Spaho et al. demonstrated that HC can enhance solution quality when applied as a refinement phase after an initial population-based optimization [28]. Similarly, Xhafa et al. applied HC across various spatial distributions, such as uniform, normal, and Weibull, reporting improvements in network structure with minimal computational overhead [29]. Furthermore, hybridizing HC with evolutionary algorithms has shown success in other network optimization tasks  [30,31].

**Figure 4:**
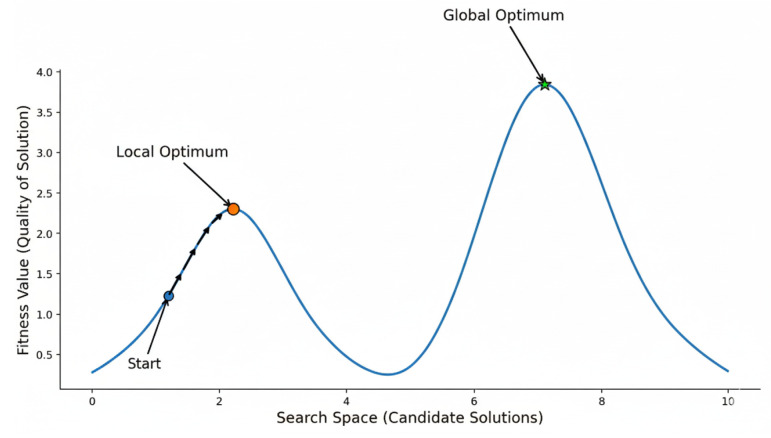
Trajectory of HC algorithm converging to a local optimum.

However, a significant drawback of HC is its tendency to become trapped at local optima. As illustrated in Figure 4, the algorithm may converge to a peak that is not the highest point on the fitness landscape because it lacks the mechanism to accept temporary setbacks (downhill moves) that might allow it to traverse a valley toward the global optimum. Consequently, while HC is excellent for local refinement, it is most effective when hybridized with global search methods that can avoid premature stagnation.

### 3.3. Distributed Genetic Algorithm (DGA)

**Figure 5:**
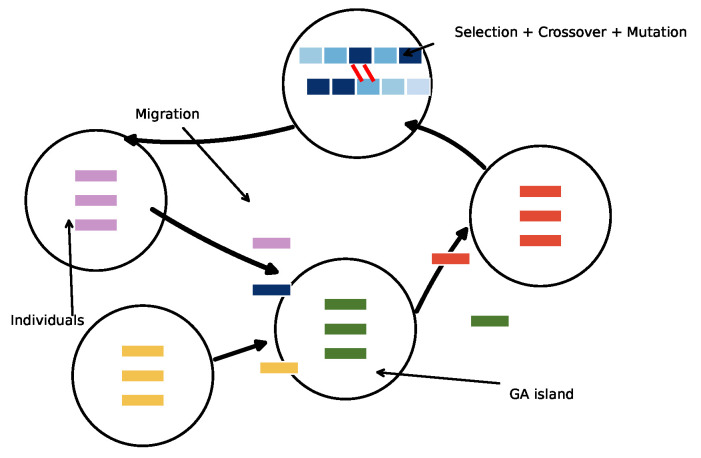
Illustration of DGA employing the island model.

The Distributed Genetic Algorithm (DGA), employing the Island Model, is a parallel evolutionary optimization strategy that addresses the limitations of GAs. In this framework, the entire population is divided into several semi-isolated subpopulations, or “islands,” each evolving independently. This isolation maintains genetic diversity by enabling different islands to explore distinct regions of the search space concurrently before sharing information as shown in Figure 5 [32].

Within each island, the optimization process employs a GA, which simulates natural selection to iteratively evolve candidate solutions. The iterative process for each island is as follows.

1. Initialization: A local subpopulation is initialized with random candidate solutions.
2. Selection: Individuals are selected for reproduction based on their fitness scores. Mechanisms such as Roulette Wheel or Tournament selection are employed to favor high-quality solutions [33].
3. Crossover: Selected parents undergo recombination to produce offspring. This is the primary exploration operator, combining genetic information from parents to discover new solutions.
4. Mutation: Random perturbations are applied to offspring with a low probability to maintain local diversity and prevent stagnation.
5. Migration: In a process distinct from the DGA, a migration operator is executed every $G_{mig}$ generations. A set of elite individuals is selected from a source island and transferred to a destination island according to a predefined topology. These migrants replace low-fitness individuals in the target island, injecting superior genetic traits and fostering global convergence [16,34].

The crossover method is the core engine of exploration in real-coded GAs. To handle the continuous search space of WSAN actor placement, we investigate four crossover methods: UNDX, SPX, BLX-α and psBLX.

#### 3.3.1. Unimodal Normal Distribution Crossover (UNDX)

UNDX is a real-coded crossover operator designed to preserve the statistical characteristics of the population [14]. UNDX generates offspring that follow the distribution defined by the parents, making it highly effective for optimization problems with strong variable dependencies.

**Figure 6:**
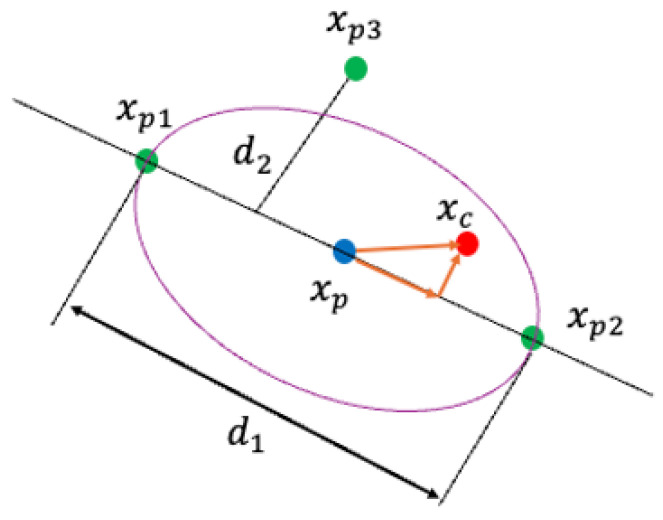
Geometric representation of UNDX showing the primary search axis and orthogonal variance.

To generate an offspring solution $x_{c}$, UNDX utilizes three parent solutions, denoted as $x_{p1}$, $x_{p2}$, and $x_{p3}$, as illustrated in Figure 6. The process begins by selecting two parents, $x_{p1}$ and $x_{p2}$, to define the primary search axis, where the vector difference is given by $d=x_{p2}-x_{p1}$. The third parent, $x_{p3}$, is then utilized to determine the spread of the search in directions orthogonal to this primary axis; specifically, the perpendicular distance $d_{2}$ from $x_{p3}$ to the line connecting the first two parents establishes the variance width. Finally, the offspring solution $x_{c}$ is generated around the midpoint $x_{p}$ of the primary parents by combining a component along the primary axis with components along the orthogonal basis vectors $e_{i}$.

The offspring vector is computed by Equation (5):

(5) $$x_{c}=x_{p}+\xi d+\sum_{i=1}^{n-1}\eta_{i}e_{i}d_{2}.$$

The parameters are defined as follows: $x_{p}=\frac{1}{2}\left(x_{p1}+x_{p2}\right)$ is the midpoint of the two main parents; $d=x_{p2}-x_{p1}$ represents the difference vector defining the primary search direction; $d_{2}$ is the perpendicular distance from the third parent $x_{p3}$ to the primary search axis; $e_{i}$ are the orthonormal basis vectors orthogonal to the primary axis *d*; ξ is a random number sampled from a normal distribution $N(0,\sigma_{\xi }^{2})$; and $\eta_{i}$ are random numbers sampled from a normal distribution $N(0,\sigma_{\eta }^{2})$. By using this formulation, UNDX explores the search space by concentrating samples along the valleys linking promising solutions while maintaining the appropriate diversity orthogonal to them.

#### 3.3.2. Simplex Crossover (SPX)

**Figure 7:**
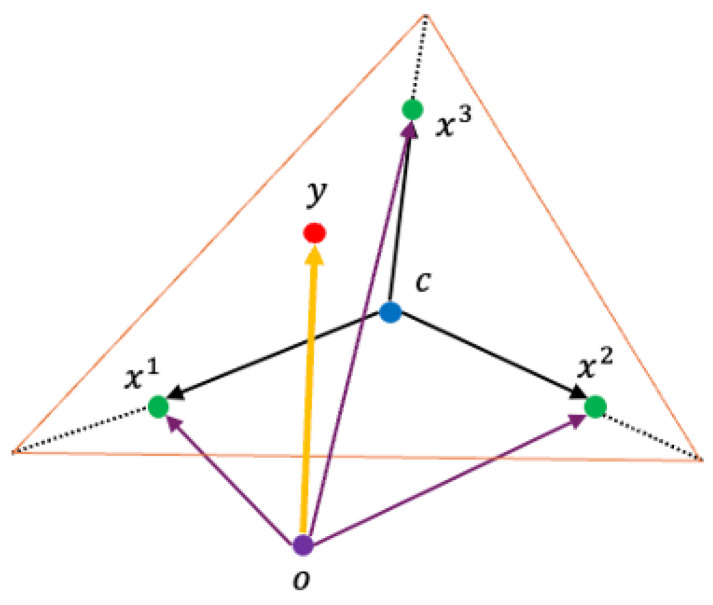
Geometric representation of SPX illustrating simplex expansion around the centroid.

SPX is a multi-parent recombination operator that exploits the geometric properties of a simplex to perform exploration. SPX generates offspring that are independent of the coordinate system. By forming a simplex using *m* parent vectors and expanding it, SPX can uniformly sample new candidate solutions from a region that adapts to the spatial distribution of the parents as illustrated in Figure 7.

To generate an offspring solution *y*, the method selects *m* parent solutions, denoted as $x^{1},x^{2},\ldots ,x^{m}$. These parents form a simplex in the solution space. The procedure first calculates the centroid of these parents. The offspring is then produced by expanding the simplex relative to this centroid by a scaling factor ϵ.

The SPX calculates the centroid *c* of the *m* parents by Equation (6):

(6) $$c=\frac{1}{m}\sum_{i=1}^{m}x^{i}.$$

The offspring vector *y* is generated using Equation (7).

(7) $$y=c+\sum_{i=1}^{m}\epsilon_{i}\gamma_{i}\left(x^{i}-O\right)$$

The parameters in Equation (7) are defined as follows:

- $x^{i}$ is the vector of the *i*th parent.
- *c* is the centroid of the selected parents.
- *O* is the reference origin for the vector operations.
- $\epsilon_{i}$ are random numbers governing the expansion, sampled uniformly to ensure diverse offspring generation.
- $\gamma_{i}$ are scaling factors that determine the extent of the simplex expansion.

#### 3.3.3. Blend Crossover (BLX-α)

BLX-α is a real-coded genetic algorithm crossover method that generates offspring by uniformly sampling within an extended region between two parent solutions [13]. BLX-α promotes diversity by expanding the search interval for each gene.

**Figure 8:**
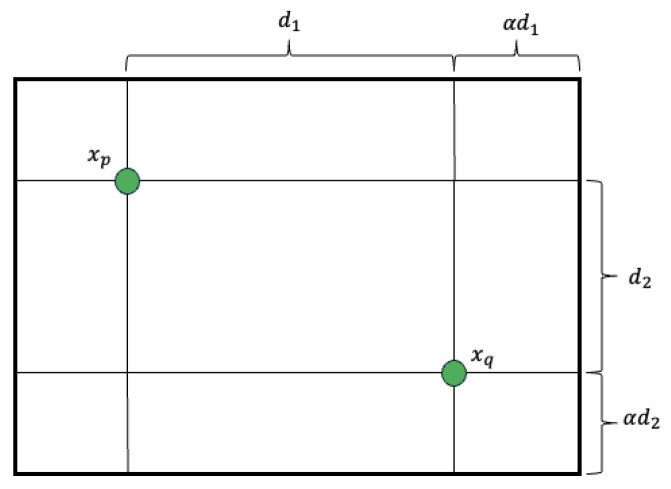
Sampling region of BLX-α defined by extending the hyper-rectangle between the parents.

As illustrated in Figure 8, let $x_{p}=\left(x_{p1},x_{p2}\right)$ and $x_{q}=\left(x_{q1},x_{q2}\right)$ represent two parent solution vectors in a two-dimensional space. The rectangular sampling region is formed by extending the distance between each gene by a factor of α. The difference along each dimension is calculated by Equation (8):

(8) $$d_{1}=x_{q1}-x_{p1},\;d_{2}=x_{q2}-x_{p2}.$$

To generate an offspring solution, uniform random values $r_{1}$ and $r_{2}$ are drawn from the range [−α,1+α]. The offspring coordinates ($x_{c1},x_{c2}$) are then computed using Equation (9):

(9) $$x_{c1}=x_{p1}+r_{1}\cdot d_{1},\;x_{c2}=x_{p2}+r_{2}\cdot d_{2}.$$

By choosing α=0.5, the offspring can be generated anywhere within a box that extends 50% beyond the range defined by the parents, ensuring a balance between exploitation (staying near parents) and exploration (searching new areas).

#### 3.3.4. Parallelotope-Shaped Blend Crossover (psBLX)

**Figure 9:**
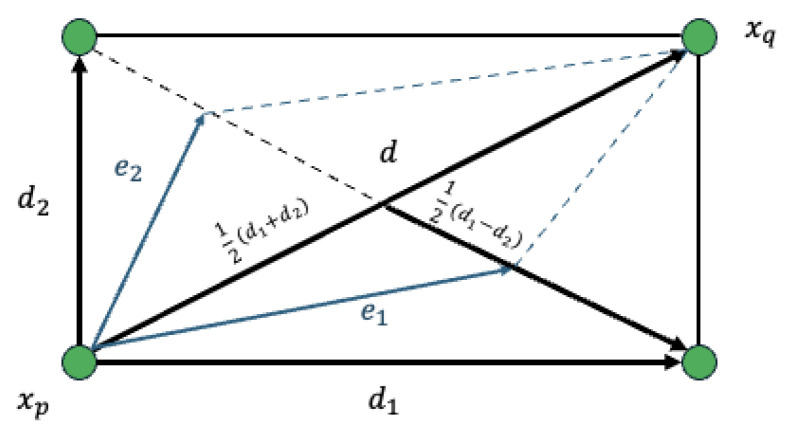
Sampling region of psBLX illustrating directional basis vectors derived from parent positions.

psBLX is an advanced real-coded crossover operator proposed by Sakai and Takahama to overcome the limitations of standard axis-aligned crossovers such as BLX-α [17]. Recent studies have also explored other real-coded operators [35,36]. While BLX-α generates offspring within a hyper-rectangle, psBLX samples offspring solutions from a parallelotope-shaped region (Figure 9). This geometric adaptation ensures that the distribution of offspring vectors reflect the spatial correlation and directionality of the parent vectors.

To generate an offspring vector, psBLX utilizes two parent vectors, $x_{p}$ and $x_{q}$, which define the diagonal vertices of the parallelotope. The geometry of the sampling region is determined relative to the difference vector $\vec{d}=x_{q}-x_{p}$. From this direction, basis vectors $e_{j}$ are constructed to define the slanted edges of the sampling region. In a two-dimensional space with component differences $d_{1}$ and $d_{2}$, these basis vectors are calculated using a shape parameter β (where β∈[0,1]) that adjusts the skew of the parallelotope. Index wrapping is applied such that $d_{D+1}=d_{1}$. The basis vectors are defined by Equation (10):

(10) $$e_{j}\left(\beta \right)=\frac{1+\beta }{2}\cdot d_{j}+\frac{1-\beta }{2}\cdot d_{j+1}.$$

The offspring vector $X_{c}$ is generated by summing random contributions along these basis vectors. A random coefficient $r_{j}$ is sampled uniformly from the range [−α,1+α] for each dimension, and the final position is computed using Equation (11):

(11) $$X_{c}=x_{p}+\sum_{j=1}^{D}r_{j}\cdot e_{j}.$$

By aligning the search range with the trajectory between parents, psBLX maintains linear correlations among parameters.

## 4. PSO-HC-DGA Hybrid System

The actor placement problem in WSANs presents a high-dimensional and non-linear search space with many local optima. This complexity makes it challenging for single-metaheuristic algorithms to balance exploration and exploitation. To overcome these limitations, we propose the PSO-HC-DGA system, which is a hybrid intelligent system that combines Particle Swarm Optimization (PSO), Hill Climbing (HC) and the Distributed Genetic Algorithm (DGA). Furthermore, hybrid systems have been applied to WMN router placement [31,37,38].

In the PSO-HC-DGA system, the total population of candidate solutions (actor placement configurations) is divided into $N_{islands}$ semi-isolated subpopulations. Each island functions as an independent optimization unit, executing a specific algorithm to evolve its local population.

The hybridization strategy is designed as follows:

- Global Exploration (PSO & GA Islands): The islands are divided into PSO-based islands and GA-based islands. PSO islands employ swarm intelligence to converge towards promising regions of the search space, while GA islands utilize crossover and mutation operators to maintain genetic diversity and prevent the system from becoming trapped at local optima.
- Local Refinement (HC): To overcome the lack of fine-tuning capabilities in standard PSO, HC is embedded within the PSO islands. After particles update their positions, HC is triggered to perform a localized search around the new position by moving only if it improves the fitness.
- Information Sharing (Migration): To ensure cooperative learning, a migration mechanism is implemented. This mechanism exchanges elite individuals between islands at fixed intervals. This prevents isolated islands from stagnating and facilitates the spread of superior genetic traits across the entire system.

### 4.1. Algorithmic Flow

**Figure 10:**
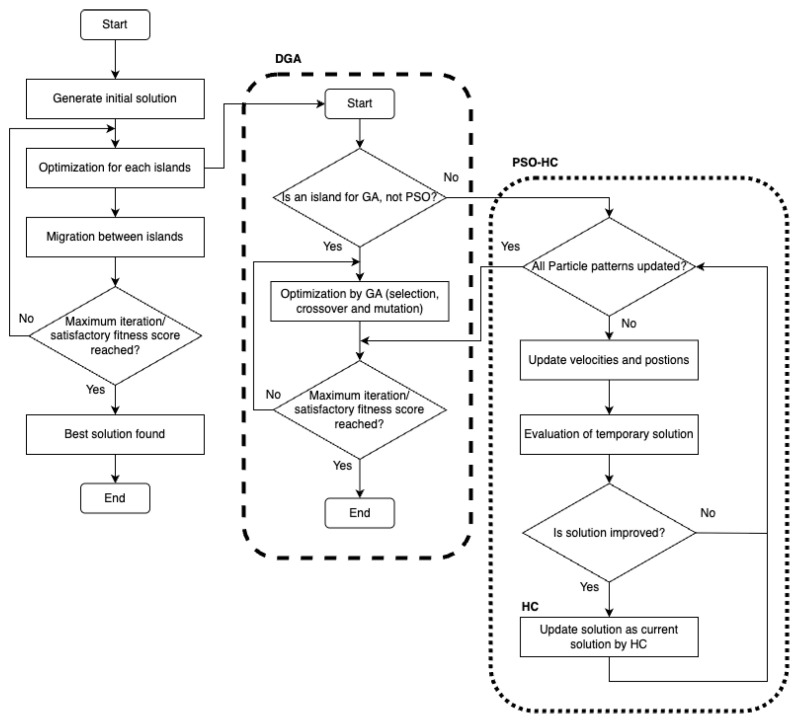
Flowchart of the proposed PSO-HC-DGA hybrid system.

The execution flow of the PSO-HC-DGA framework is illustrated in Figure 10 and proceeds as follows:

1. Initialization: A global population of $N_{pop}$ individuals is generated. Each individual represents a complete set of coordinates for all *M* actors in the WSAN, $A=\{\left(x_{1},y_{1}\right),\ldots ,\left(x_{M},y_{M}\right)\}$. These individuals are distributed evenly among the islands.
2. Island Evolution Loop: For each generation, the islands evolve in parallel.
  - Within GA Islands: Genetic operators (Selection, Crossover, Mutation) are applied. Crossover methods generate offspring by combining actor coordinates from parent solutions.
  - Within PSO Islands:
    (a) *Velocity and Position Update:* Particles update their velocities and positions using PSO equations, incorporating actor replacement methods. These updates are guided by their personal best ($P_{best}$) and the island’s global best ($G_{best}$).
    (b) *HC Refinement:* For each particle, HC is executed. The actor positions are perturbed slightly; if the new configuration yields a higher fitness, the particle is updated to this refined position.
3. Migration Phase: Every $G_{mig}$ generations, a migration operator is triggered. The best-performing individual (elite) from a source island is copied to a destination island, replacing the worst-performing individual on the destination island.
4. Termination: The process repeats until the maximum number of iterations is reached or the fitness score value is maximized.

The pseudo-code for the proposed system is presented in Algorithm 1.
**Algorithm 1** Pseudocode of the PSO-HC-DGA Hybrid System1:**Input:** Number of islands $N_{islands}$, Migration interval $G_{mig}$, Max iterations $G_{max}$2:**Output:** Global Best Solution $G_{best}$3:**Initialize** global population and distribute into $N_{islands}$ subpopulations $P_{k}$4:Calculate fitness for all individuals5:**while** Current Iteration $<G_{max}$ **do**6:    **for** each Island k=1 to $N_{islands}$ **do**7:        **if** Island *k* is **GA Type then**8:            **while** Local GA condition not met **do**9:                **Selection:** Select parents from $P_{k}$10:              **Crossover:** Apply UNDX, SPX, BLX-α, or psBLX11:              **Mutation:** Apply boundary mutation12:              **Update:** Evaluate offspring and replace worst individuals in $P_{k}$13:          **end while**14:      **end if** Island *k* is **PSO Type then**15:          **while** All Particles in $P_{k}$ not updated **do**16:              **for** each Particle *i* **do**17:                  **PSO Update:** Calculate $v_{i}^{t+1}$ and $x_{i}^{t+1}$18:                  Evaluate Fitness $F(x_{i}^{t+1})$19:                  *// Hill Climbing Refinement*20:                  Generate neighbor solution $x_{i}^{\prime }$ by local perturbation21:                  **if** $F\left(x_{i}^{\prime }\right)>F\left(x_{i}^{t+1}\right)$ **then**22:                      $x_{i}^{t+1}\leftarrow x_{i}^{\prime }$ {Accept HC improvement}23:                  **end if**24:                  Update Personal Best ($P_{best}$) and Local Best ($L_{best}$)25:              **end for**26:          **end while**27:      **end if**28:  **end for**29:  **if** Current Iteration $G_{mig}=0$ **then**30:     **Migration:** Select elite individuals from source islands and transfer to destination islands31:  **end if**32:  Update Global Best Solution $G_{best}$ across all islands33:  Increment Iteration34:**end while**34:**return** $G_{best}$

### 4.2. Fitness Function

The evaluation of a candidate solution (actor placement configuration) is governed by a fitness function that balances three conflicting objectives: maximizing network connectivity, maximizing sensor coverage, and ensuring efficient actor utilization (load balancing). The proposed fitness function is a weighted sum of these three metrics calculated by Equation (12),

(12) F=α·SGC+β·NCS+γ·ASA,

subject to the following constraint (Equation (13)):

(13) α+β+γ=1.

These metrics are defined as follows.

- **SGC (Size of the Giant Component):** This metric evaluates the actor–actor connectivity. It represents the number of actors contained in the largest connected component of the actor network graph $G_{A}$. The range of SGC is 0≤SGC≤|A|, where |A| is the total number of actors. A value of |A| indicates that the entire actor network is connected.
- **NCS (Number of Covered Sensors):** This metric measures the coverage performance. It counts the total number of sensor nodes that are within the communication range of at least one actor. The range is 0≤NCS≤|S|, where |S| is the total number of sensors. Maximizing NCS ensures that data from the maximum number of sensors can be collected.
- **ASA (Average Sensors per Actor):** This metric provides load balancing. It is calculated by Equation (14):
  (14) $$\mathrm{ASA}=\frac{\mathrm{NCS}}{\left|A\right|}.$$
  The range of ASA is $0\leq \mathrm{ASA}\leq \frac{\left|S\right|}{\left|A\right|}$. This term encourages configurations where actors are placed in areas with a high density of sensors.
  *Standard Deviation (SD) of the sensor load.* To evaluate the load balancing of sensors among actors, let $C_{a}$ represent the number of sensors connected to actor a∈A. The Standard Deviation (SD) of the sensor load is quantified by Equation (15):
  (15) $$\mathrm{SD}\;=\;\sqrt{\frac{1}{\left|A\right|}\sum_{a\in A}{\left(C_{a}-\mathrm{ASA}\right)}^{2}}.$$
  A small SD indicates a balanced assignment (most actors have similar numbers of sensors), whereas large SD reveals uneven load where a few actors have many sensors and others have few.

## 5. Evaluation Results

In this section, we present the simulation results of the proposed PSO-HC-DGA hybrid system for the optimal placement of actors in WSANs. We evaluate the performance of four crossover methods (UNDX, SPX, BLX-α, and psBLX) combined with two actor replacement methods (RIWM and FC-RDVM). The simulations were conducted using the parameters outlined in Table 1. We consider three scenarios: small-scale, medium-scale, and large-scale WSANs. The instances were selected to match previous works [29,39,40]. The number of simulations is 100, and the number of migrations is 300. For evaluation, we calculate the average values. We assign the highest priority for SGC because this is the most important metric related with WSAN connectivity. We consider the number of covered sensors (NCS) the second most important metric. The last metric is ASA, which is related to load balancing. We carried out many simulations with different ratios (8:1:1, 7:2:1, 6:3:1, and 5:4:1), but the best performance was for the ratio **6:3:1**.

### 5.1. Small-Scale Scenario (16 Actors, 48 Sensors)

#### 5.1.1. Actor Connectivity and Sensor Coverage for Small-Scale Scenario

**Figure 11:**
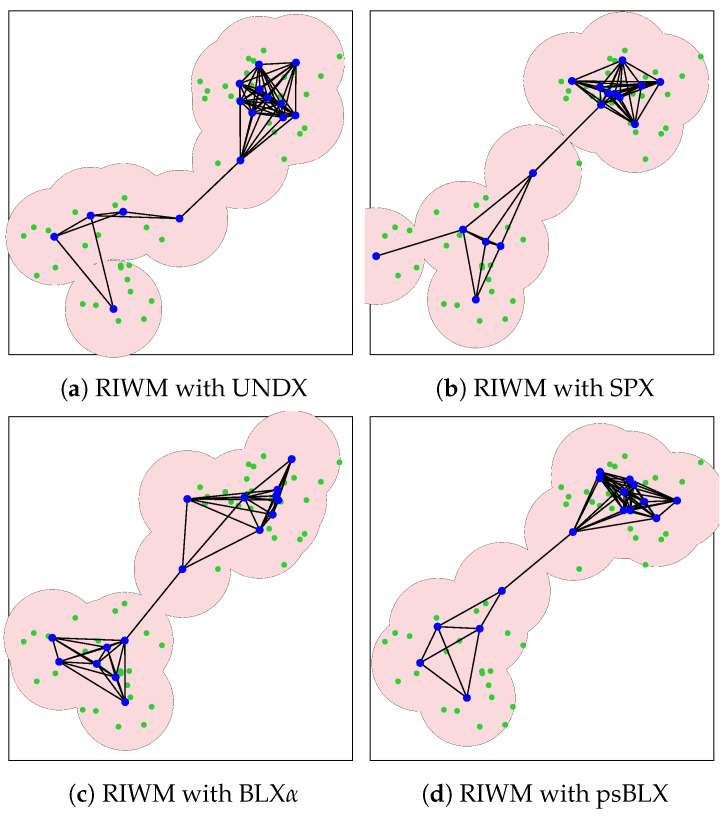
Visualization of optimized network deployment for the small-scale scenario with RIWM.

**Figure 12:**
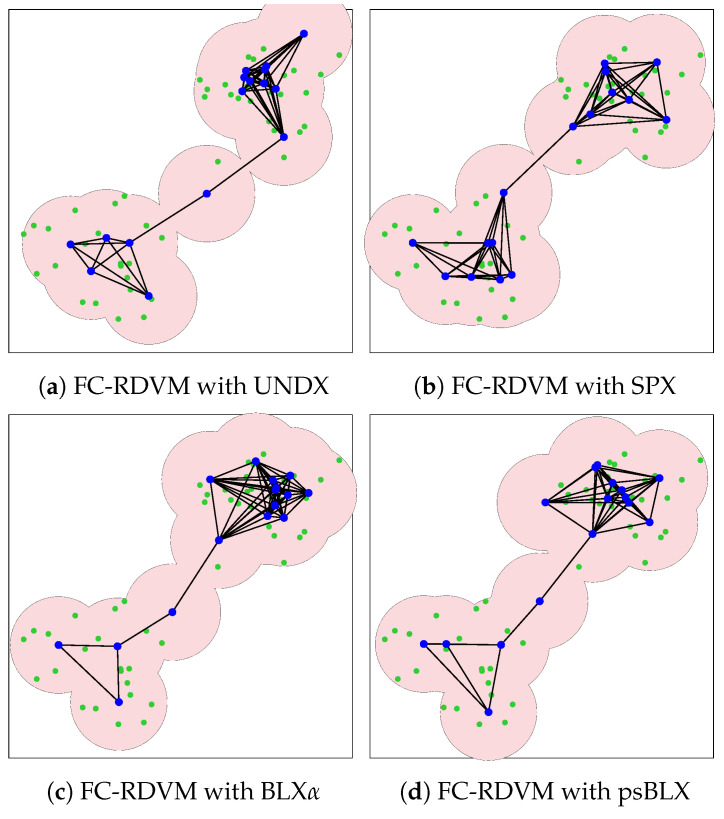
Visualization of optimized network deployment for the small-scale scenario with FC-RDVM.

In the small-scale scenario, the network comprises 48 sensors (green points) distributed into two distinct clusters and managed by 16 actors (blue points). The pink circles surrounding each mesh router represent the signal coverage area. The simulation results for actor connectivity (SGC) and sensor coverage (NCS) demonstrated that all tested configurations achieved optimal performance. As shown in visualization results (Figure 11 and Figure 12), every combination of crossover methods and actor replacement methods achieved 100% connectivity (SGC = 16) and 100% coverage (NCS = 48). The actors formed a “bridge” connecting the two separated sensor zones, a behavior driven by the high priority (α=0.6) assigned to connectivity in the fitness function. These results suggest that for this node density, all four crossover methods find optimal bridge positions.

#### 5.1.2. Load Balancing for Small-Scale Scenario

Beyond connectivity and coverage, the efficiency of the WSAN is determined by how evenly the sensing load is distributed among the actors (load balancing). We evaluate this using the SD of sensors per actor; a lower value indicates better load balancing.

**Figure 13:**
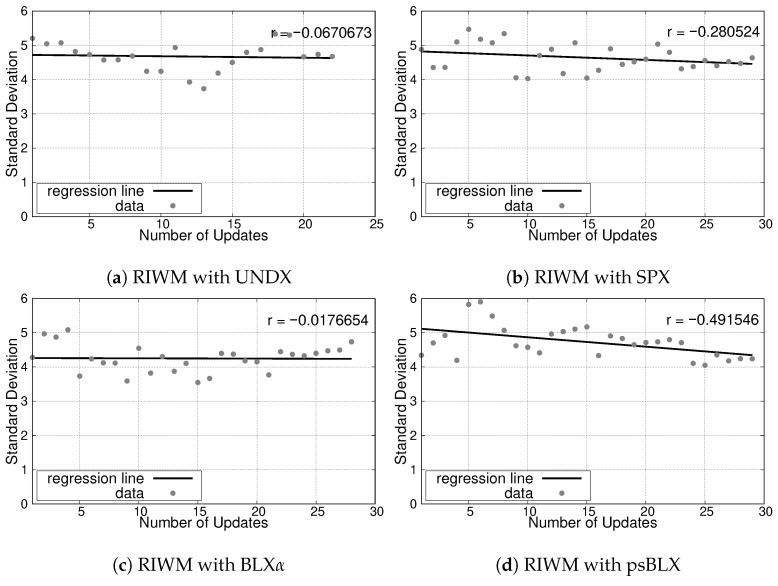
Load balancing analysis for the small-scale scenario: SD vs. number of updates for RIWM.

**Figure 14:**
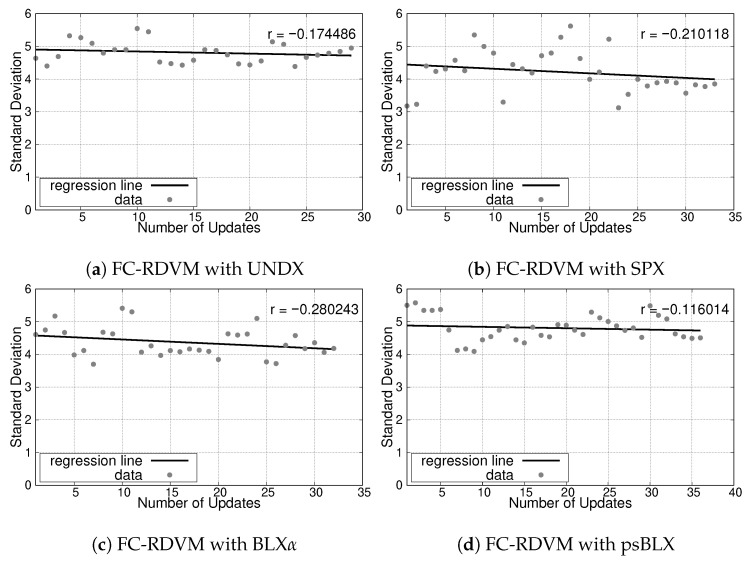
Load balancing analysis for the small-scale scenario: SD vs. number of updates for FC-RDVM.

The simulation results for the small-scale scenario, illustrating the relationship between the number of updates and the SD, are presented in Figure 13 for RIWM and Figure 14 for FC-RDVM. The correlation coefficient (*r*) serves as an indicator of the convergence trend toward a balanced load.

For **RIWM**, the crossover methods showed variation in performance. UNDX resulted in minimal changes in SD with a correlation coefficient of r≈−0.06 (Figure 13a). SPX achieved a decreasing SD trend with r≈−0.28 (Figure 13b). BLX-α showed a flat trend with a correlation of r≈−0.01 (Figure 13c). Finally, psBLX achieved better load balancing with a strong negative correlation of r≈−0.49 (Figure 13d).

For **FC-RDVM**, a decreasing trend was found across methods. UNDX achieved a correlation coefficient of r≈−0.17 (Figure 14a). SPX performed slightly better, with r≈−0.21 (Figure 14b). BLX-α had better load balancing in this group with a correlation coefficient r≈−0.28 (Figure 14c), while psBLX showed a smaller decrease in SD than other methods with r≈−0.12 (Figure 14d).

In the small-scale scenario, all combination reached 100% actor connectivity and sensor coverage (SGC and NCS), while, for load balancing, **RIWM** combined with **psBLX** had better performance.

To confirm that these observed differences were statistically significant and not due to randomness, we performed the Kruskal–Wallis H-test on SD values. We selected this non-parametric test because it does not assume that the simulation data follow a normal distribution. The results are summarized in Table 2.

The test results confirm that the choice of crossover methods affects performance. For RIWM, the test returned a *p*-value of $5.70\times 10^{-4}$. This proves that the variations in load balancing are statistically significant. The results for FC-RDVM were stronger, showing a higher H-statistic (31.48) and a lower *p*-value ($6.73\times 10^{-7}$). Since a higher H-statistic indicates a greater divergence between the groups, this suggests that there is performance differences between crossover methods.

### 5.2. Medium-Scale Scenario (32 Actors, 96 Sensors)

#### 5.2.1. Actor Connectivity and Sensor Coverage for Medium-Scale Scenario

In the medium-scale scenario, the network consists of 96 sensors (green points) distributed into two distinct clusters and managed by 32 actors (blue points). The simulation results for SGC and NCS revealed that while connectivity remained robust, significant disparities emerged in coverage performance. All tested configurations achieved 100% connectivity (SGC = 32), successfully maintaining the bridge between zones. However, coverage performance varied.

**Figure 15:**
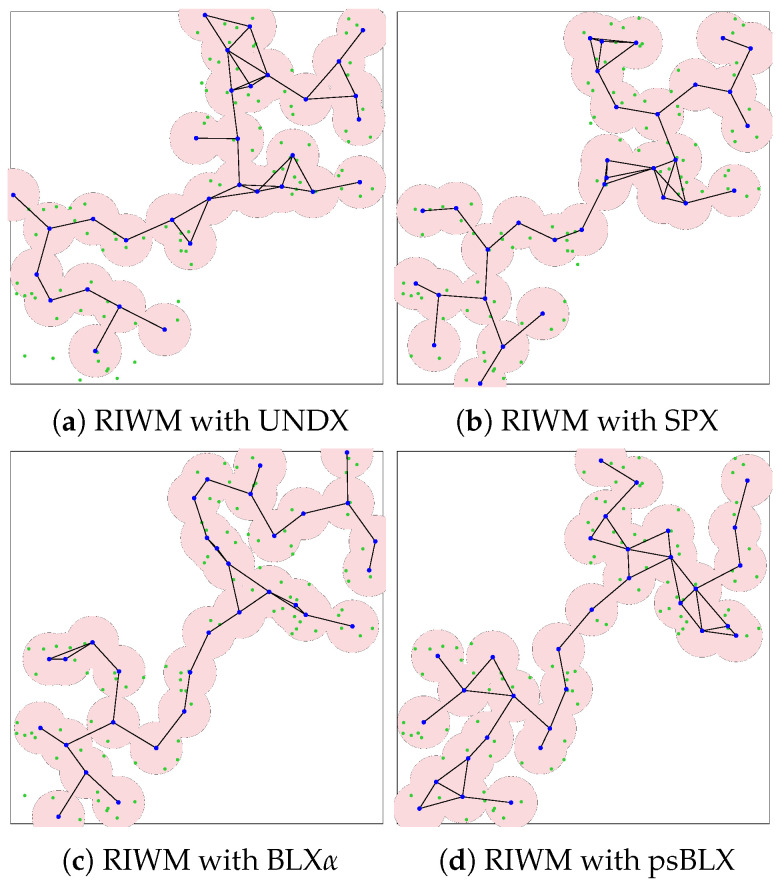
Visualization of optimized network deployment for the medium-scale scenario with RIWM.

As shown by the visualized results in Figure 15, RIWM combined with UNDX (Figure 15a) covered 88 sensors. This was improved by SPX (Figure 15b) and BLX-α (Figure 15c), which reached 94 and 95 sensors, respectively, while psBLX (Figure 15d) performed best, with 100% coverage (NCS = 96).

**Figure 16:**
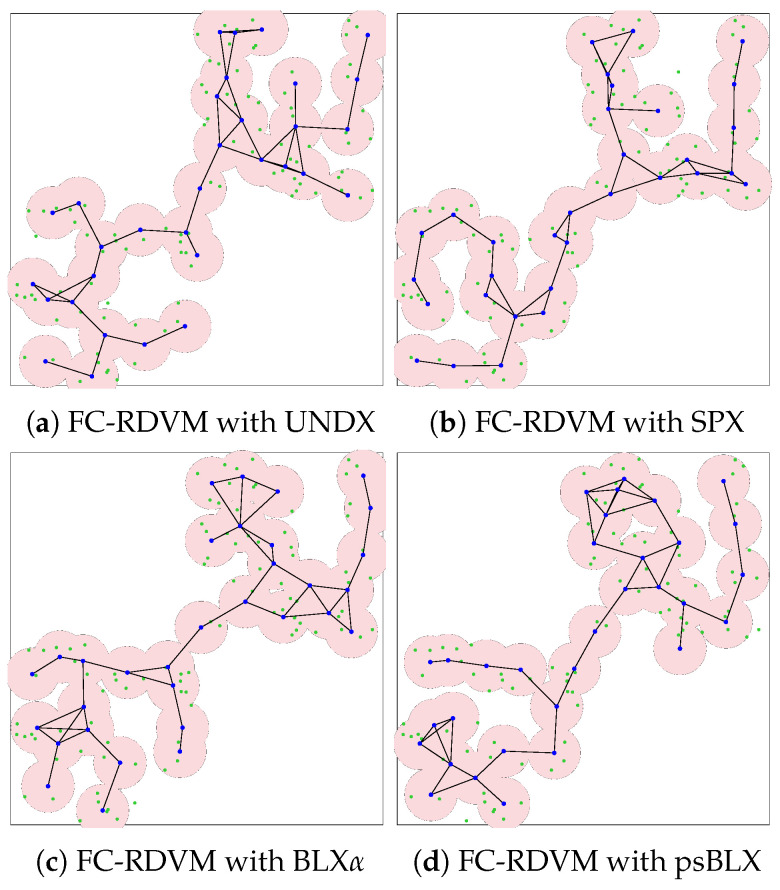
Visualization of optimized network deployment for the medium-scale scenario with FC-RDVM.

For FC-RDVM (Figure 16), the results showed incomplete coverage. UNDX (Figure 16a) and SPX (Figure 16b) each covered 94 sensors. BLX-α (Figure 16c) performed slightly better in this group, achieving coverage of 95 sensors. In addition, psBLX (Figure 16d) covered 94 sensors. These results indicates that for higher node density, the search space becomes more challenging.

In the medium-scale scenario, all combinations achieved 100% actor connectivity (SGC = 32). Regarding sensor coverage, the **RDVM** combined with **psBLX** achieved full sensor coverage (NCS = 96), while **FC-RDVM** combined with **BLX-α** had the best sensor coverage in the FC-RDVM group, with 95 covered sensors.

**Figure 17:**
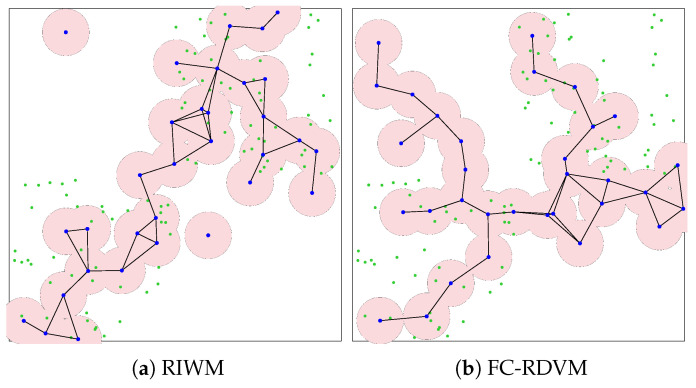
Visualization of optimized network deployment for the medium-scale scenario optimized by PSO.

**Figure 18:**
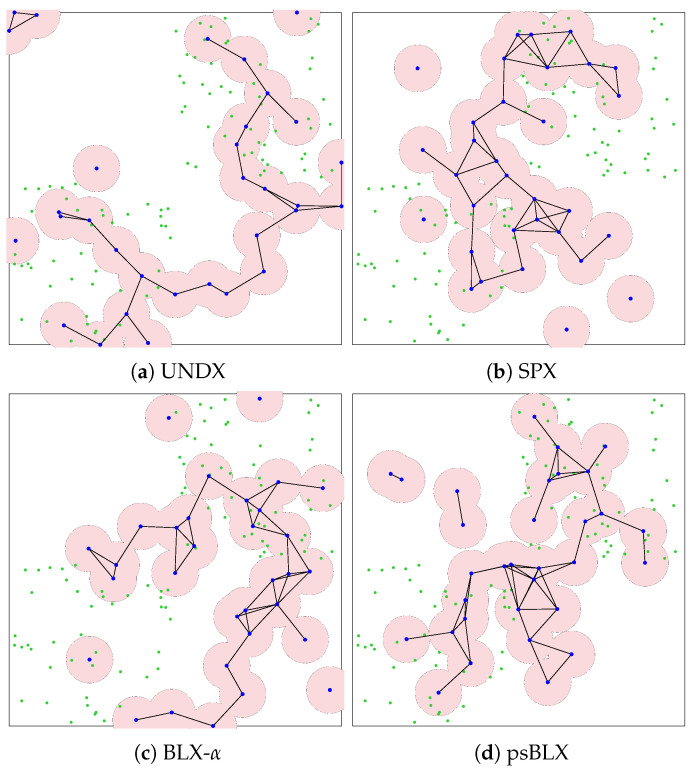
Visualization of optimized network deployment for the medium-scale scenario optimized by GA.

For the medium-scale scenario, we show the visualization results of the PSO system (Figure 17) and the GA system (Figure 18). The PSO system managed to maintain connectivity but failed to all cover sensors, leaving coverage holes visible in the outer regions of the clusters. Similarly, the GA system showed scattered actor placements, failing to connect all actors.

In the small-scale scenario, all methods reached optimal results very quickly with no difference. However, the medium-scale scenario is more complex, which helps to reveal the performance differences.

**Figure 19:**
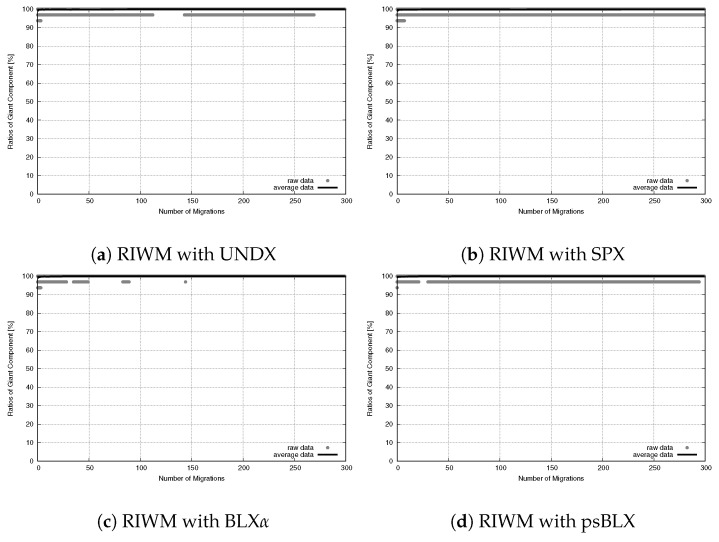
Connectivity evolution for medium-scale WSANs in the case of RIWM.

**Figure 20:**
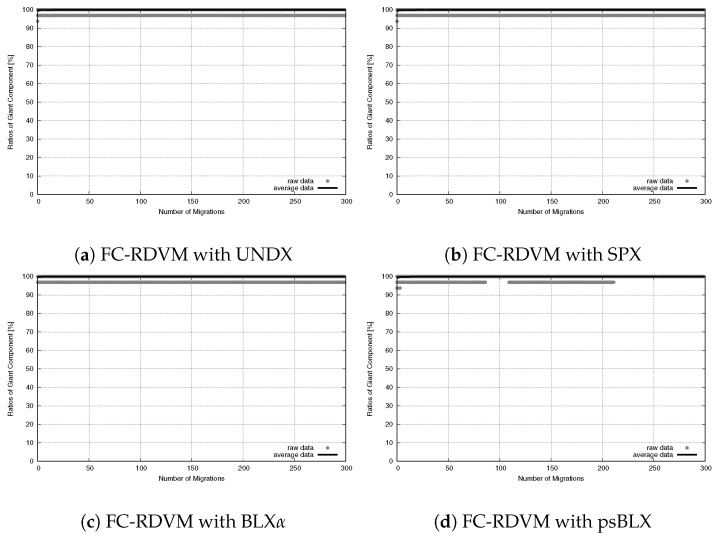
Connectivity evolution for medium-scale WSANs in the case of FC-RDVM.

To understand the network stability, we tracked the SGC ratio over 300 migration steps. As shown in Figure 19 for RIWM and Figure 20 for FC-RDVM, the SGC ratio stayed at 100% from the start to the end. This result confirms that the high priority given to connectivity in the fitness function worked well. It kept the actors from breaking the main connection between the two zones while they tried to improve other goals.

**Figure 21:**
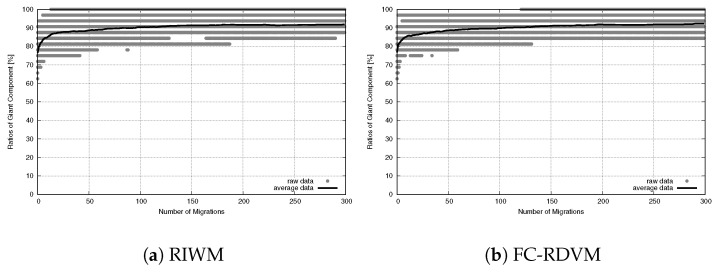
Connectivity evolution for medium-scale WSANs optimized by PSO.

**Figure 22:**
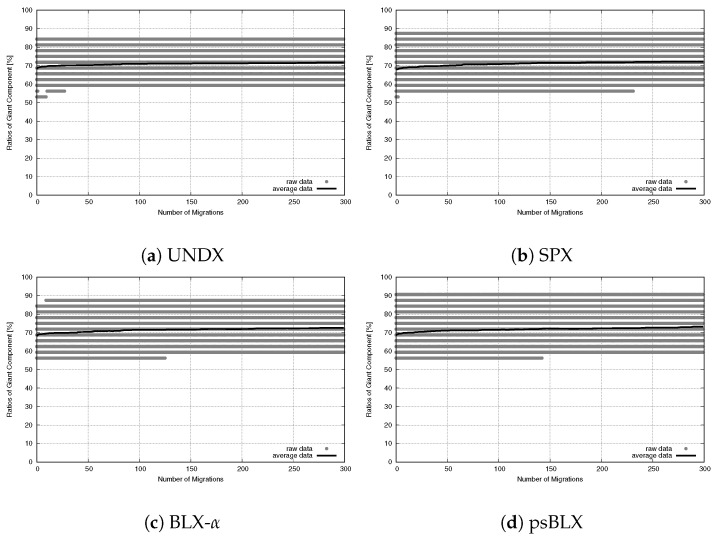
Connectivity evolution for medium-scale WSANs optimized by GA.

We compared the PSO-HC-DGA hybrid system with a PSO system. As shown in Figure 21, the PSO system exhibited a wider variance, reflecting high instability and a strong tendency to stagnate in local optima. Additionally, we evaluated a GA system (Figure 22). Unlike PSO-based methods, which use velocity for fine-tuning, the GA relies on stochastic operations, which resulted in lower SGC. This confirms that the velocity component of our hybrid system is essential for maintaining the good actor positioning required for robust connectivity.

**Figure 23:**
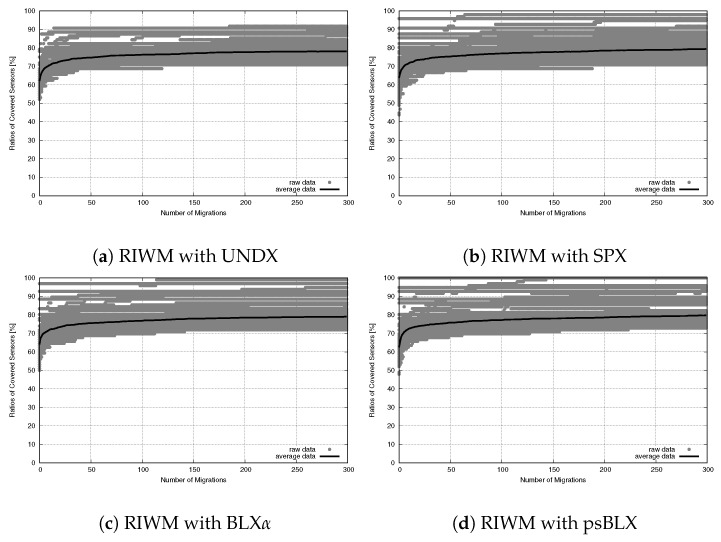
Sensor coverage convergence for medium-scale RIWM: ratio of covered sensors vs. migration steps.

**Figure 24:**
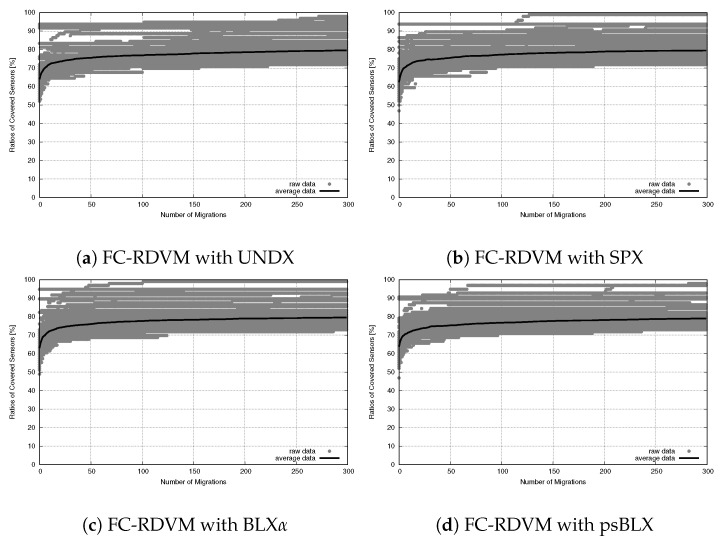
Sensor coverage convergence for medium-scale FC-RDVM: ratio of covered sensors vs. migration steps.

While connectivity was stable, the sensor coverage (NCS) had differences in performance, as shown in the convergence trends for RIWM (Figure 23) and FC-RDVM (Figure 24).

For RIWM, the performance varied between methods. UNDX (Figure 23a) showed premature convergence. Although the narrow gray area indicated that UNDX combined with RIWM was stable, it failed to reach 100% coverage. SPX (Figure 23b) and BLX-α (Figure 23c) showed a much wider gray area that extended to the top of the graph. This indicates instability: while some runs reached optimal coverage, others performed poorly. Furthermore, psBLX (Figure 23d) showed a wider gray area than UNDX, but it rapidly reached 100% coverage (NCS = 96) in the early stages, achieving more optimal performance.

FC-RDVM increased the initial speed for all methods and the differences in performance between the crossover methods, are smaller. Unlike RIWM, the results for FC-RDVM showed comparable convergence trends. UNDX (Figure 24a), SPX (Figure 24b), BLX-α (Figure 24c) and psBLX (Figure 24d) demonstrates premature convergence. This suggests that the high initial velocity became the main factor, limiting the search and causing the swarm to remain at local optima.

**Figure 25:**
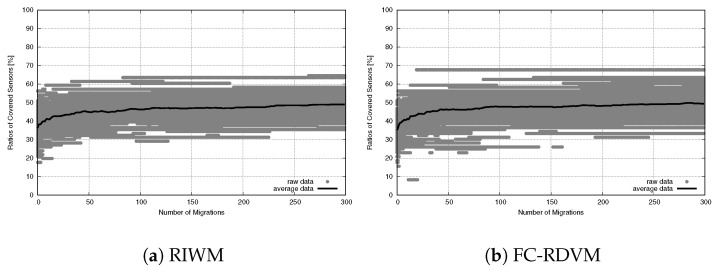
Sensor coverage convergence for medium-scale WSANs optimized by PSO.

**Figure 26:**
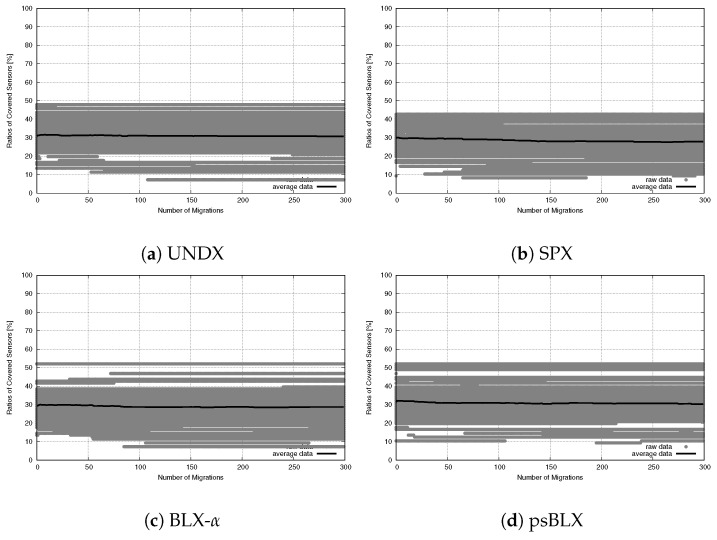
Sensor coverage convergence for medium-scale WSANs optimized by GA.

To validate the effectiveness of our proposed hybrid system, we carried out simulations for the medium-scale scenario with PSO- and GA-based systems considering NCS. As illustrated Figure 25, the PSO system failed to reach 100% of NCS for both the RIWM and FC-RDVM actor replacement methods. The PSO system showed lower coverage compared to the optimal hybrid system, confirming that standard velocity updates alone are insufficient to resolve the coverage challenges in two-zone topologies. Similarly, the GA system (Figure 26) exhibited a slower convergence rate. These results demonstrate that PSO-HC-DGA combines the fine-tuning of PSO with the exploration of GA and outperforms the PSO and GA systems.

#### 5.2.2. Load Balancing for Medium-Scale Scenario

For the medium-scale scenario, network complexity increases, with 32 actors servicing 96 sensors. This scenario tests the algorithm’s ability to fine-tune solutions in a larger search space.

**Figure 27:**
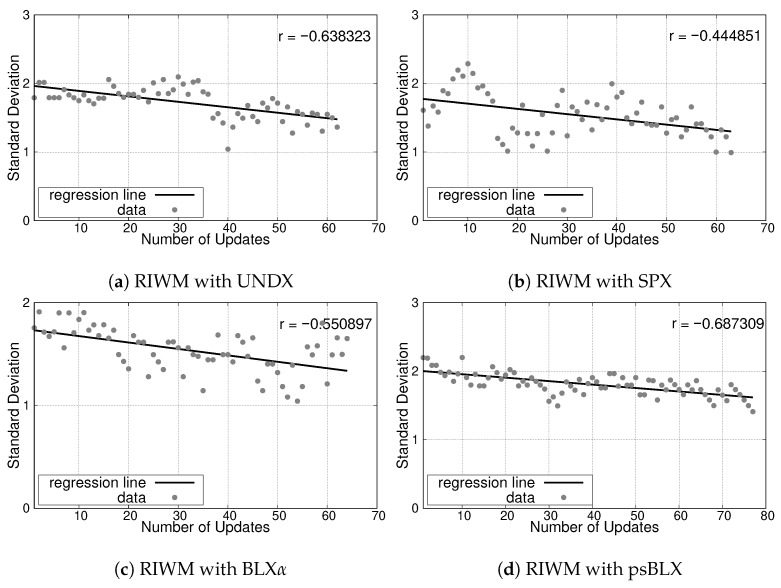
Load balancing analysis for the medium-scale scenario: SD vs. number of updates for RIWM.

**Figure 28:**
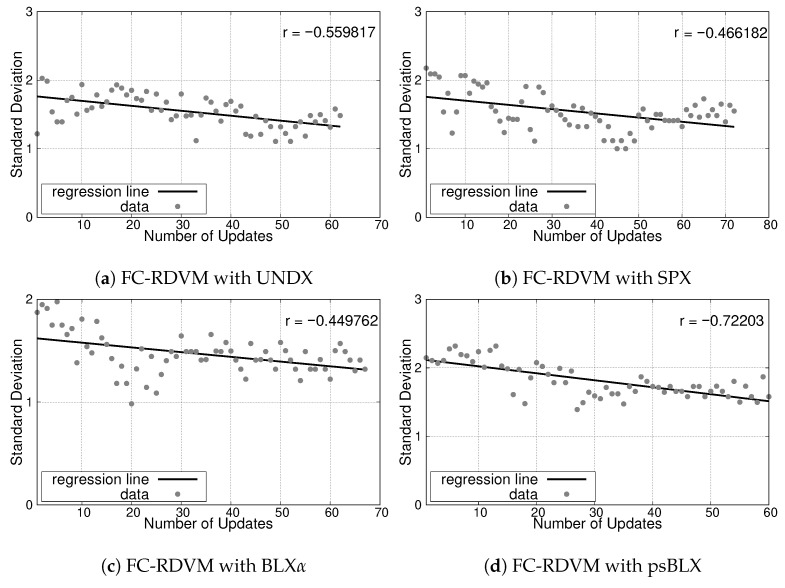
Load balancing analysis for the medium-scale scenario: SD vs. number of updates for FC-RDVM.

The analysis of load balancing (reflected by the SD) showed different performance trends compared to the small-scale scenario. The negative slopes observed in the regression lines suggest that the optimization process attains success by balancing the workload among actors. The results are presented in Figure 27 for RIWM and Figure 28 for FC-RDVM.

For **RIWM**, most methods showed improved convergence at the medium scale compared to the small scale scenario. UNDX achieved good performance, with r≈−0.64 (Figure 27a). SPX showed moderate performance, with r≈−0.44 (Figure 27b). BLX-α had better load balancing than SPX, with r≈−0.55 (Figure 27c). With a strong negative correlation of r≈−0.69, psBLX achieved better load balancing than the other crossover methods (Figure 27d).

The correlation coefficients for **FC-RDVM** combined with UNDX and SPX are r≈−0.55 (Figure 28a) and r≈−0.46 (Figure 28b), respectively. BLX-α showed the smallest improvement in this group (r≈−0.44, Figure 28c), while psBLX had the best performance, with a correlation coefficient of r≈−0.72 (Figure 28d), demonstrating the most efficient method for load balancing in this scenario.

In the medium-scale scenario, **psBLX** was the most effective crossover method for load balancing. Furthermore, **FC-RDVM combined with psBLX** had the best overall performance.

We performed the Kruskal–Wallis H-test on the medium-scale results to statistically evaluate the differences between crossover methods. The test statistics are presented in Table 3.

The results showed even stronger statistical significance than those form the small-scale scenario. Both replacement methods produced *p*-values far below the standard threshold ($p\approx 10^{-13}$ and $p\approx 10^{-12}$), confirming that the variations in solution quality were statistically significant.

Importantly, comparing these results to the small-scale scenario reveals the impact of network scale. The H-statistics in this medium-scale scenario (H≈61.66 for RIWM) were double those recorded in the small-scale scenario (H≈17.45). Since a higher H-statistic indicates a larger divergence among the methods, this marked increase demonstrates that as the problem complexity grows (more actors, larger search space), the choice of crossover method becomes increasingly critical.

### 5.3. Large-Scale Scenario (64 Actors, 192 Sensors)

In the large-scale scenario, the complexity of the network increases significantly, with 192 sensors (green points) distributed across the two zones and managed by 64 actors (blue points). To evaluate the scalability of the proposed system, we selected the optimal configuration identified in the medium-scale scenario, PSO-HC-DGA hybrid system (FC-RDVM combined with psBLX), and compared it with a PSO system (FC-RDVM).

The simulation results revealed a contrast in performance scalability. The PSO-HC-DGA hybrid system successfully maintained 100% actor connectivity (SGC = 64 actors) and achieved high sensor coverage (NCS = 186 sensors), demonstrating that the combination between PSO’s exploitation and GA’s exploration remains effective in high-dimensional search spaces.

**Figure 29:**
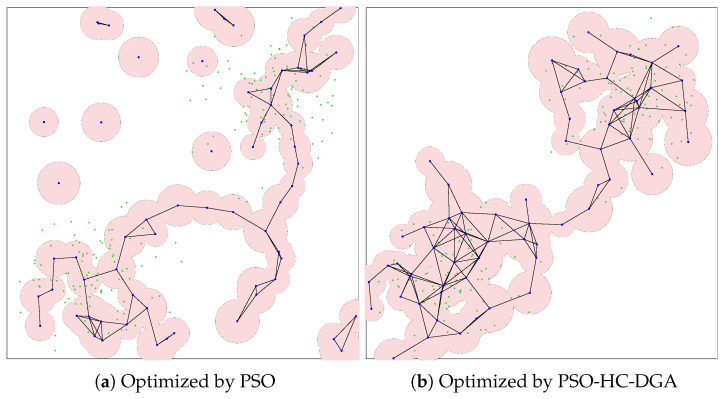
Visualization of optimized network deployment for the large-scale scenario.

In contrast, the PSO system struggled significantly at this scale. While it managed to maintain a connected backbone in some runs, it failed to provide adequate coverage for the peripheral sensors. As illustrated in the visualization results (Figure 29), the deployment optimized by the PSO system (Figure 29a) exhibited a fragmented and sparse topology. Instead of forming a unified network, the actors remained in isolated clusters, leaving vast portions of the sensor field uncovered. This weakness aligns with the connectivity results, where the PSO system failed to integrate all actors into a single component, while the PSO-HC-DGA hybrid system (Figure 29b) formed a robust and mesh-like backbone. The actors distributed themselves to create multiple redundant paths, effectively spanning the entire region and maximizing sensor coverage while ensuring a fully connected network.

**Figure 30:**
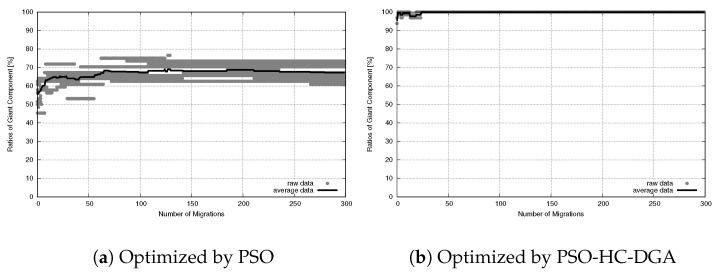
Connectivity evolution for large-scale WSANs.

The results presented in Figure 30 show the connectivity under the two systems. As shown in Figure 30a, the PSO system failed to establish a fully connected network. The SGC ratio stagnated at approximately 70%, with a wide variance that reflected instability. This confirmed that in the large-scale scenario, the velocity update was unable to overcome the local optima associated with the complex, disconnected search space, leaving the network permanently fragmented.

The PSO-HC-DGA system (Figure 30b) demonstrated rapid and robust convergence. The system achieved 100% connectivity in the very early stages of the simulation and maintained this stability throughout the entire process. This result demonstrates that the hybrid system’s diversity mechanisms prevent fragmentation, ensuring a reliable communication backbone even as the network scale increases.

**Figure 31:**
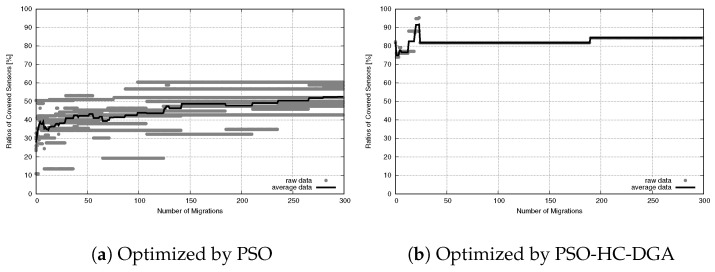
Sensor coverage convergence for large-scale WSANs.

The limitations of the PSO system became even more apparent when we analyzed the sensor coverage performance. Figure 31 plots the evolution of the NCS over 300 migrations.

For the PSO system (Figure 31a), the results indicated a severe failure to explore the search space. The average coverage started low and rose gradually, but it reached a maximum of only 50%. The extremely wide gray area represented high variance, meaning that the algorithm’s performance was unpredictable and highly dependent on random initialization. These quantitative data correlate directly with the visualization results (Figure 29a), confirming that the swarm was trapped at local optima and could not spread out to cover the peripheral sensors.

The PSO-HC-DGA system (Figure 31b) showed superior search capability. It rapidly converged to a high coverage ratio of approximately 85% within the first few iterations and maintained this level with negligible variance. The flat, stable trajectory indicates that the hybrid system successfully prevented the swarm from stagnating, allowing the actors to distribute themselves effectively across the expanded 192-sensor field.

## 6. Conclusions

This study presented the PSO-HC-DGA hybrid system, which is a hybrid system that integrates PSO, HC and DGA to optimize actor placement in WSANs. The proposed system addresses the NP-hard challenge of achieving strong actor connectivity, full sensor coverage and load balancing. We considered three metrics, namely, SGC, NCS and ASA, to define the fitness function for evaluating the quality of each placement configuration. The key findings from the simulation results were as follows:

- **Small-Scale Scenario (16 Actors)**
  − **Actor Connectivity (SGC):** All tested combinations of crossover and actor replacement methods achieved complete actor connectivity (SGC = 16).
  − **Sensor Coverage (NCS):** Full sensor coverage (NCS = 48) was successfully achieved for all configurations.
  − **Load Balancing (ASA):** Significant differences were observed for load balancing. The combination of **RDVM** and **psBLX** achieved superior load balancing.
- **Medium-Scale Scenario (32 Actors)**
  − **Actor Connectivity (SGC):** All configurations maintained complete actor connectivity, consistently achieving SGC = 32.
  − **Sensor Coverage (NCS):** Sensor coverage performance varied based on the crossover method.
    * **RIWM Results:** The combination of **RIWM** with **psBLX** achieved complete sensor coverage (NCS = 96).
    * **FC-RDVM Results:** The combination of **FC-RDVM** and **BLX-α** had the best coverage within its group, covering 95 sensors.
  − **Load Balancing (ASA): psBLX** proved to be the most effective crossover method for both actor replacement methods. Specifically, **FC-RDVM** combined with **psBLX** demonstrated better load balancing than the other combinations.
- **Large-Scale Scenario (64 actors)**
  − **Actor Connectivity (SGC):** The PSO-HC-DGA hybrid system achieved 100% actor connectivity (SGC = 64), confirming that the connectivity-prioritized fitness function remained effective even as the network scale increased.
  − **Sensor Coverage (NCS):** The PSO-HC-DGA hybrid system achieved 99% sensor coverage (NCS = 190), demonstrating that the proposed system effectively scaled to high-density environments.

The statistical significance of performance differences in load balancing was confirmed using the Kruskal–Wallis H-test (*p* < 0.05). The increased H-statistics in the medium-scale scenario indicate that as network scale and complexity grow, the selection of the appropriate crossover method becomes increasingly critical for system performance.

Furthermore, comparative simulations demonstrated the advantage of the PSO-HC-DGA hybrid system over simple PSO and GA systems. PSO exhibited high instability and a tendency to become trapped at local optima, while the GA suffered from slow convergence. The proposed hybrid system effectively overcame these limitations by combining the fast exploitation of PSO with the diversity preservation of DGA.

In future work, we plan to enhance the proposed system by incorporating emerging optimization algorithms, such as Beluga Whale Optimization (BWO) and Cat Swarm Optimization (CSO). We will also examine alternative crossover and actor replacement methods to further improve the balance between exploration and exploitation. 

## Figures and Tables

**Table 1:** Simulation parameters and values.

Parameters	Values
Number of Actors	16, 32, 64
Number of Sensors	48, 96, 192
Number of Simulations	100
Number of GA Islands	16
Number of Migrations	300
Number of Evolution Steps	9
Mutation Method	Boundary Mutation
Selection Method	Random Method
Crossover Methods	UNDX, SPX, BLX-α and psBLX
Actor Replacement Methods	RIWM and FC-RDVM
Topology	Two-zone industrial environment
α:β:γ	6:3:1

**Table 2:** Kruskal–Wallis H-test results for the small-scale scenario.

Replacement Method	H-Statistic	*p*-Value
RIWM	17.4536	$5.70\times 10^{-4}$
FC-RDVM	31.4819	$6.73\times 10^{-7}$

Note: The test compares the SD distributions across the four crossover methods (UNDX, SPX, BLX-α, psBLX).

**Table 3:** Kruskal–Wallis test results for the medium-scale scenario.

Replacement Method	H-Statistic	*p*-Value
RIWM	61.6557	$2.60\times 10^{-13}$
FC-RDVM	58.3182	$1.34\times 10^{-12}$

Note: The high H-statistics compared to the small-scale results indicate that method selection is critical in larger networks.

## Data Availability

The data presented in this study are available upon request from the corresponding author because of privacy and ownership considerations.

## Abbreviations

The following abbreviations are used in this manuscript:

WSANs	Wireless Sensor and Actor Networks
IIoT	Industrial Internet of Things
APP	Actor Placement Problem
PSO	Particle Swarm Optimization
GA	Genetic Algorithm
DGA	Distributed Genetic Algorithm
HC	Hill Climbing
UNDX	Unimodal Normal Distribution Crossover
SPX	Simplex Crossover
BLX-α	Blend Crossover
psBLX	Parallelotope-Shaped Blend Crossover
RIWM	Random Inertia Weight Method
FC-RDVM	Fast Convergence Rational Decrement of $V_{max}$ Method
SGC	Size of the Giant Component
NCS	Number of Covered Sensors
ASA	Average Sensors per Actor
SD	Standard Deviation

## References

[1] Wollschlaeger M., Sauter T., Jasperneite J. (2017). The future of industrial communication: Automation networks in the era of the internet of things and industry 4.0. IEEE Ind. Electron. Mag..

[2] Sisinni E., Saifullah A., Han S., Jennehag U., Gidlund M. (2018). Industrial internet of things: Challenges, opportunities, and directions. IEEE Trans. Ind. Inform..

[3] Akyildiz I.F., Kasimoglu I.H. (2004). Wireless sensor and actor networks: Research challenges. Ad Hoc Netw..

[4] Melodia T., Pompili D., Gungor V.C., Akyildiz I.F. (2007). Communication and coordination in wireless sensor and actor networks. IEEE Trans. Mob. Comput..

[5] Abbasi A.A., Younis M.F., Baroudi U.A. (2013). Recovering from a node failure in wireless sensor-actor networks with minimal topology changes. IEEE Trans. Veh. Technol..

[6] Younis M., Akkaya K. (2008). Strategies and techniques for node placement in wireless sensor networks: A survey. Ad Hoc Netw..

[7] Alaiwy M., Alaiwy F., Habib S. (2007). Optimization of Actors Placement Within Wireless Sensor-Actor Networks. Proceedings—IEEE Symposium on Computers and Communications, Santiago, Portugal, 1-4 July 2007.

[8] Che N., Li Z., Jiang S. (2010). Actor Deployment Strategies in WSANs. 2010 First International Conference on Pervasive Computing, Signal Processing and Applications, Harbin, China, 17–19 September 2010.

[9] Zeng H., Kang Z. (2017). Relay node placement to restore connectivity in wireless sensor networks. 2017 IEEE 9th International Conference on Communication Software and Networks (ICCSN), Guangzhou, China, 6–8 May 2017.

[10] Kennedy J., Eberhart R. Particle swarm optimization. Proceedings of the ICNN’95—International Conference on Neural Networks.

[11] Shi Y., Eberhart R. Parameter selection in particle swarm optimization. Proceedings of the 7th International Conference on Evolutionary Programming VII.

[12] Deb K., Agrawal R.B. (1995). Simulated binary crossover for continuous search space. Complex Syst..

[13] Eshelman L.J., Schaffer J.D. (1993). Real-Coded Genetic Algorithms and Interval-Schemata. Foundations of Genetic Algorithms 2.

[14] Kita H., Ono I., Kobayashi S. Theoretical analysis of the unimodal normal distribution crossover for real-coded genetic algorithms. Proceedings of the 1998 IEEE International Conference on Evolutionary Computation.

[15] Tsutsui S., Yamamura M., Higuchi T. Multi-parent recombination with simplex crossover in real coded genetic algorithms. Proceedings of the 1st Annual Conference on Genetic and Evolutionary Computation.

[16] Whitley D., Rana S., Heckendorn R.B. The Island Model Genetic Algorithm: On Separability, Population Size and Convergence. Proceedings of the International Conference on Computer and Information Technology.

[17] Sakai S., Takahama T. (2023). Improving a Real-Coded Genetic Algorithm Using an Outward Vector Rate and Parallelotope-Shaped Crossover. RIMS Kôkyûroku.

[18] Lu C., Saifullah A., Li B., Sha L., Gonzalez H., Gunatilaka D., Wu C., Nie L., Chen Y., Jayachandran P. (2016). Real-time wireless sensor–actuator networks for industrial control systems. Proc. IEEE.

[19] Vitturi S., Zunino C., Sauter T. (2019). Industrial communication systems and their future challenges: Next-generation Ethernet, IIoT, and 5G. Proc. IEEE.

[20] Seferagić A., Famaey J., De Poorter E., Hoebeke J. (2020). Survey on wireless technology trade-offs for the industrial internet of things. Sensors.

[21] Periyanayagi S., Kulandaivel R., Susikala S. (2011). Placement of actors in wireless sensor networks. International Conference on Advanced Computer Technology.

[22] Mohammadi S., Farahani G. (2020). Computational intelligence-based connectivity restoration in wireless sensor and actor networks. EURASIP J. Wirel. Commun. Netw..

[23] Olariu S., Stojmenovic I. (2006). Design Guidelines for Maximizing Lifetime and Avoiding Energy Holes in Sensor Networks with Uniform Distribution and Uniform Reporting. IEEE Trans. Mob. Comput..

[24] Gungor V.C., Hancke G.P. (2009). Industrial Wireless Sensor Networks: Challenges, Design Principles, and Technical Approaches. IEEE Trans. Ind. Electron..

[25] Clerc M., Kennedy J. (2002). The particle swarm-explosion, stability, and convergence in a multidimensional complex space. IEEE Trans. Evol. Comput..

[26] Shi Y., Eberhart R.C. A modified particle swarm optimizer. Proceedings of the 1998 IEEE International Conference on Evolutionary Computation Proceedings.

[27] Eberhart R.C., Shi Y. Comparing inertia weights and constriction factors in particle swarm optimization. Proceedings of the 2000 Congress on Evolutionary Computation.

[28] Spaho E., Xhafa A., Elmazi D., Xhafa F., Barolli L. A Study on Performance of Hill Climbing Heuristic Method for Router Placement in Wireless Mesh Networks. Proceedings of the 12th International Conference on Network-Based Information Systems (NBiS).

[29] Xhafa F., Sánchez C., Barolli L. Locals Search Algorithms for Efficient Router Nodes Placement in Wireless Mesh Networks. Proceedings of the 12th International Conference on Network-Based Information Systems (NBiS).

[30] Oda T., Barolli A., Spaho E., Barolli L., Xhafa F. (2013). Genetic algorithms for node placement in WMNs: Effect of changes in population size and number of generations. J. High Speed Netw..

[31] Barolli A., Xhafa F., Takizawa M., Barolli L. (2014). Performance evaluation of WMN-GA system for different settings of population size and number of generations. Hum. Cent. Comput. Inf. Sci..

[32] Gong Y., Fukunaga A. Distributed Island-Model Genetic Algorithms Using Heterogeneous Parameter Settings. Proceedings of the 2011 IEEE Congress on Evolutionary Computation.

[33] Goldberg D.E. (1989). Genetic Algorithms in Search, Optimization, and Machine Learning.

[34] Alba E., Troya J.M. (1999). A survey of parallel distributed genetic algorithms. Complexity.

[35] Deep K., Thakur M. (2007). A new crossover operator for real coded genetic algorithms. Appl. Math. Comput..

[36] Herrera F., Lozano M., Verdegay J.L. (2003). A taxonomy for the crossover operator for real-coded genetic algorithms: An experimental study. Int. J. Intell. Syst..

[37] Sakamoto S., Oda T., Ikeda M., Barolli L. (2015). Design and Implementation of a Simulation System Based on Particle Swarm Optimization for Node Placement Problem in Wireless Mesh Networks. 2015 International Conference on Intelligent Networking and Collaborative Systems, Taipei, Taiwan, 2–4 September 2015.

[38] Chang X., Oda T., Spaho E., Ikeda M., Barolli L., Xhafa F. (2013). Performance analysis of WMNs using hill climbing algorithm for different distributions of mesh clients. 2013 Seventh International Conference on Complex, Intelligent, and Software Intensive Systems, Taichung, Taiwan, 3–5 July 2013.

[39] Xhafa F., Sánchez C., Barolli L. (2012). Local search methods for efficient router nodes placement in wireless mesh networks. J. Intell. Manuf..

[40] Xhafa F., Sánchez C., Barolli L. (2010). Tuning Operators of Genetic Algorithms for Mesh Routers Placement Problem in Wireless Mesh Networks. 2010 International Conference on Broadband, Wireless Computing, Communication and Applications, Fukuoka, Japan, 4–6 November 2010.

